# Oral Consumption of Bread from an RNAi Wheat Line with Strongly Silenced Gliadins Elicits No Immunogenic Response in a Pilot Study with Celiac Disease Patients

**DOI:** 10.3390/nu13124548

**Published:** 2021-12-18

**Authors:** María H. Guzmán-López, Susana Sánchez-León, Miriam Marín-Sanz, Isabel Comino, Verónica Segura, Luis Vaquero, Octavio M. Rivero-Lezcano, Jorge Pastor, Carolina Sousa, Santiago Vivas, Francisco Barro

**Affiliations:** 1 Department of Plant Breeding, Institute for Sustainable Agriculture—Spanish National Research Council (IAS-CSIC), 14004 Córdoba, Spain; mhguzman@ias.csic.es (M.H.G.-L.); ssanchez@ias.csic.es (S.S.-L.); mmarin@ias.csic.es (M.M.-S.); 2 Department of Microbiology and Parasitology, Faculty of Pharmacy, University of Seville, 41012 Sevilla, Spain; icomino@us.es (I.C.); vsegura@us.es (V.S.); csoumar@us.es (C.S.); 3 Department of Gastroenterology, University Hospital of León, University of León, 24071 León, Spain; luisvaqueroayala@gmail.com (L.V.); svivasa@gmail.com (S.V.); 4 Research Unit, University Hospital of León, University of León, 24071 León, Spain; orivero@saludcastillayleon.es; 5 Novapan, S.L., C/Chopo, 68-70, La Puebla de Alfindén, 50171 Zaragoza, Spain; jorge-pastor@panishop.com

**Keywords:** short-term oral challenge, RNAi wheat, 33-mer, gluten-free diet, celiac, E82, gliadins, gluten

## Abstract

Celiac disease (CD) is a genetically predisposed, T cell-mediated and autoimmune-like disorder caused by dietary exposure to the storage proteins of wheat and related cereals. A gluten-free diet (GFD) is the only treatment available for CD. The celiac immune response mediated by CD4+ T-cells can be assessed with a short-term oral gluten challenge. This study aimed to determine whether the consumption of bread made using flour from a low-gluten RNAi wheat line (named E82) can activate the immune response in DQ2.5-positive patients with CD after a blind crossover challenge. The experimental protocol included assessing IFN-γ production by peripheral blood mononuclear cells (PBMCs), evaluating gastrointestinal symptoms, and measuring gluten immunogenic peptides (GIP) in stool samples. The response of PBMCs was not significant to gliadin and the 33-mer peptide after E82 bread consumption. In contrast, PBMCs reacted significantly to Standard bread. This lack of immune response is correlated with the fact that, after E82 bread consumption, stool samples from patients with CD showed very low levels of GIP, and the symptoms were comparable to those of the GFD. This pilot study provides evidence that bread from RNAi E82 flour does not elicit an immune response after a short-term oral challenge and could help manage GFD in patients with CD.

## 1. Introduction

Celiac disease (CD) is an autoimmune enteropathy caused by exposure to dietary gluten in genetically predisposed individuals. These proteins are strong environmental factors because they are resistant to complete digestion in the human digestive tract, thereby providing peptides capable of inducing an immune response through the human leukocyte antigen (HLA) system when HLA-DQ2 or HLA-DQ8 haplotypes are present [1]. In patients with CD, the recognition of gluten peptides activates an autoimmune response mediated by CD4+ T cells, leading to small intestine inflammation, intestinal villus atrophy, malabsorption of nutrients, and derived complications [2]. The immune response is strongly enhanced because of the deamination of glutamine residues present in gluten peptides by tissue transglutaminase 2 (tTG2) in the intestinal mucosa, leading to higher binding affinity and more stable peptide–HLA–DQ complexes [3]. Although many CD-related epitopes are recognized by CD4+ T cells [4], a 33-mer peptide derived from α-gliadin, which contains six copies of three overlapping HLA DQ2.5-restricted T cell epitopes, is the main contributor to the immunogenicity of gluten [5]. Production of interferon (IFN-γ) by relevant T cells can be evaluated in peripheral blood by the enzyme-linked immunospot (ELISPOT) assay after a short-term (3 days) oral gluten challenge [6]. This approach has many applications to identify CD dominant epitopes, to uncover differences in the immune response between different cereals, and for testing new therapies for CD [7,8,9].

Permanent strict adherence to a gluten-free diet (GFD) is currently the only effective treatment for CD. A strict oral diet for life is difficult to follow because of the unintended contamination of “gluten-free” products, improper labeling, social constraints, and ubiquity of gluten proteins in raw or cooked foods and pharmaceuticals. At least 67% of the patients with CD get exposed to gluten despite best efforts at dietary modifications [10,11]. Perhaps this is a contributing reason why 25–40% of adults with CD still have villous atrophy after 2 years on a GFD [12]. In addition, many gluten-free products tend to be less healthy than their gluten analogs [13]. The development of dietary and non-dietary therapies for CD is clinically important; moreover, these therapies will help in improving the patient’s quality of life [14,15].

We have applied biotechnological approaches such as RNAi and CRISPR/Cas9 (clustered regularly interspaced short palindromic repeats/CRISPR-associated protein 9) to down-regulate or directly edit relevant gliadin genes for CD [16,17]. RNAi is a conserved eukaryotic post-transcriptional mechanism that leads to the specific degradation of target messenger RNA (mRNA), preventing its translation into protein [18,19]. RNAi response is triggered by the presence of double-stranded RNA (dsRNA) that activates a third-class ribonuclease known as Dicer, which degrades this dsRNA into small interfering RNA (siRNA) of 21–25 nucleotides in length. The presence of siRNAs activates another enzymatic complex known as RISC (RNA-Induced Silencing Complex), which uses one of the strands of these siRNAs as a template to identify complementary messenger RNA (mRNA). CRISPR/Cas9 consists of an RNA-guided nuclease that allows the introduction of small deletions in DNA following inefficient DNA repair after nuclease cleavage, resulting in gene knockout [20]. The RNAi strategy has been proven to be highly effective, as the gluten content decreases by up to 98% in these new wheat varieties [21], abolishing their ability to stimulate the T cell clones derived from the intestinal lesions in patients with CD [16] or the PBMCs in vitro assays [22]. An RNAi line (denoted as E82) produced using RNAi technology is very promising; it contains very low levels of gliadins but retains the high molecular weight glutenins related to breadmaking quality. Consequently, we hypothesized that bread made using flour from the low-gluten RNAi E82 wheat line could not elicit an immunogenic response in patients with CD. The present pilot study sought to test this hypothesis further by investigating whether the consumption of E82 bread can elicit an immune response in DQ2.5-positive patients with CD after a blind crossover gluten challenge by the analysis of peripheral T cell response. This response was compared to that elicited after a brief consumption of Standard bread.

## 2. Materials and Methods

### 2.1. Patients and In Vivo Challenge

**Figure 1:**
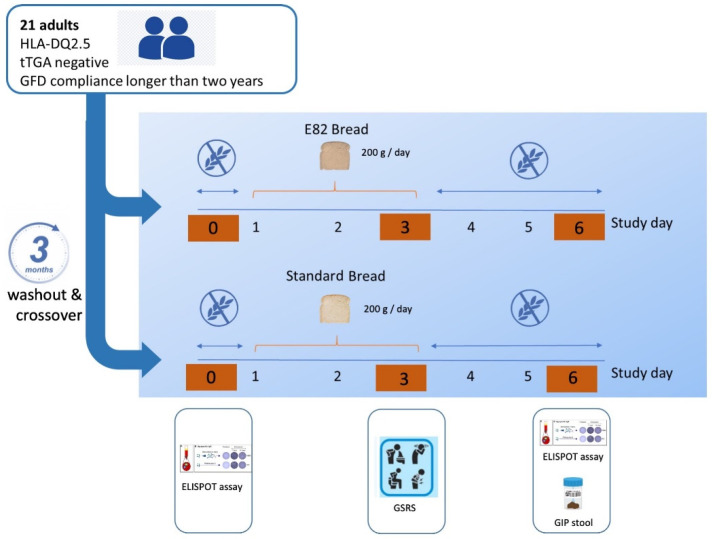
Pilot study design. The study design was a blind, randomized crossover of diet challenge. Subjects were randomized to a gluten challenge with gluten-containing bread (Standard bread) or with a bread made using flour from a low-gluten RNAi wheat line (E82 bread). Patients eat bread (200 g day) for 3 days, and after a 3-month washout period are allocated to the other bread group. Symptoms were scored at the end of the challenge by Gastrointestinal Symptom Rating Scale (GSRS). Enzyme-linked immunospot (ELISPOT) assay was determined on day 0 and day 6. GIP content was evaluated from stool samples on day 6.

Twenty-one patients diagnosed with CD were enrolled in this study based on positive serology (tissue transglutaminase antibodies (tTGA)) and villous atrophy at duodenal biopsy. All of them were positive for haplotype HLA-DQ2.5. The inclusion criteria included the following: (1) a strict GFD compliance longer than two years (range, 2–8 years), (2) negative serology (tTGA), and (3) a total recovery of their initial duodenal villous atrophy. Subjects were recruited from the outpatient gastroenterology clinic of the Hospital of León (León, Spain). Subjects were randomized to a gluten challenge with gluten-containing bread (Standard bread) or with bread made using flour from a genetically modified low-gluten wheat (E82 bread) (Figure 1). Randomization was made with a macro for the SPSS statistics program that created an initial diet for participants who were consecutively included in the diet assigned by this type of simple randomization. All subjects were instructed to eat bread (200 g/day) assigned for randomization, for a 3-day period, and then all the individuals from both groups followed a 3-month washout period and were allocated to the other bread group using the same protocol (200 g/day for 3 days). The presence of symptoms was evaluated at the end of the gluten challenge, using a questionnaire based on the Gastrointestinal Symptom Rating Scale (GSRS), which assesses the severity of gastrointestinal (GI) symptoms through a 7-point Likert scale in five clusters: indigestion, diarrhea, constipation, abdominal pain, and reflux [23].

Patients were instructed to communicate any medication taken during the study period. They were informed about the purpose of the study and had provided their full written consent. The study design was approved by the local ethics committee of the Hospital of León, Spain (approval number 1626).

### 2.2. Preparation of Bread Types

The ingredients for E82 bread were as follows: flour from a low-gluten RNAi wheat line (5.40 kg, 58.65%), water at 27 °C (3.78 kg, 41.06%), and salt (27 g, 0.29%), reaching a total of 9.207 kg. Ingredients were briefly mixed and stayed in autolysis at 26 °C for 7 h, and subsequently, gluten-free yeast (110 g) and the rest of the salt (80 g) were incorporated into the dough. The ingredients were mixed for 10 min (8 min at speed 1 and 2 min at speed 2), and the final temperature of the dough was checked (23.5 °C). After a bulk resting time of 55 min, the dough was divided into 760 g portions of dough, which were formed and introduced in sandwich bread molds and fermented in a SALVA fermentation chamber (Salva Industrial, S.L.U., Lezo, Guipúzcoa, Spain) at 30 °C and 85% humidity for 55 min. Finally, E82 bread was baked in a SALVA stone oven (Salva Industrial, S.L.U., Lezo, Guipúzcoa, Spain) at 215 °C for 40 min. After cooling, the bread was sliced, packed in bags, and frozen at −20 °C.

For Standard bread, a pre-dough was prepared the day before by mixing Rebola sourdough (545 g, 13.42%), BELL wheat flour type 550 (2.177 kg, 53.58%), water (1.265 kg, 31.13%), salt (43 g, 1.06%), and yeast (33 g, 0.81%), making a total of 4.063 kg. Pre-dough was mixed for 20 min and then rested for 21 h (2 h at room temperature, and 19 h in the refrigerator at 2 °C). This pre-dough acted as a starter culture, contributing with lactic acid bacteria and wild yeast to the acidification process during the fermentation phase. The main dough for Standard bread was as follows: pre-dough (4.063 kg, 11.43%), BELL wheat flour type 550 (19.100 kg, 53.73%), water (11.850 kg, 33.33%), yeast (190 g, 0.53%), and salt (350 g, 0.98%), making a total main dough of 35.553 kg. Ingredients were mixed for 20 min (15 min at V1, and 5 min at V2), and temperature was checked (25 °C). After a bulk resting time of 90 min, the dough was divided into 760 g pieces, formed and introduced into sandwich bread molds, and fermented in a SALVA fermentation chamber at 30 °C and 85% humidity for 90 min. Finally, Standard bread was baked in a SALVA rack oven at 220 °C for 45 min. After cooling, the bread was sliced, packed in bags, and frozen at −20 °C.

Both bread types were stored at the Hospital of León, Spain, until being supplied frozen to test subjects, for defrosting immediately before consumption. Because the study included a 3-months washout period, the bread storage time ranged from one week to 3.5 months.

### 2.3. Protein, Starch, Fructans, and Gluten Content Determination

Samples from Standard and E82 bread were weighed, freeze-dried, and re-weighed to determine the water content. The samples were then ground and analyzed. Total grain protein content was determined via Dumas methodology [24]. Starch content was determined according to the standard ICC method no. 123/1 [25]. Gluten content in parts per million (ppm) was determined at the National Centre for Biotechnology (C.N.B.-C.S.I.C.) using the RIDASCREEN^®^ Gliadin competitive (R-Biopharm AG, Darmstadt, Germany) enzyme-linked immunosorbent assays (ELISA) kit, using the monoclonal antibody R5 [26]. The assay was performed on four different samples.

The fructan content per dry weight was determined in duplicate using 150 mg of sample (white flour) by the K-FRUC kit from Megazyme (www.megazyme.com, accessed on 12 January 2021) following the manufacturer assay procedure. To express the fructan content per dry weight, the moisture of each sample was determined.

### 2.4. Detection of IFN-γ Secreting Cells in Peripheral Blood Mononuclear Cells (PBMCs) by ELISPOT

PBMCs were isolated from 5–10 mL of heparinized peripheral blood before the challenge (day 0), as well as 6 days after the beginning of the challenge (day 6), and were then assayed for antigen recognition by the IFN-γ ELISPOT assay, as previously described [7]. PBMCs were collected by gradient centrifugation using Ficoll-Histopaque (Sigma Aldrich, Madrid, Spain), and 2 × 10^5^ cells/mL were cultured in 200 μL of RPMI-1640 culture medium (Gibco, Thermo Scientific, Madrid, Spain) supplemented with 10% fetal bovine serum (Gibco, Thermo Scientific), 1% penicillin–streptomycin, and 0.1% gentamicin (Sigma-Aldrich). Gliadin from wheat (Sigma-Aldrich, G3375) was digested with pepsin (Sigma-Aldrich, P6887) and trypsin (Sigma-Aldrich, T9201). Briefly, 500 mg of gliadin was incubated in 0.2 N HCl containing 5 mg of pepsin for 4 h at 37 °C. Then, the pH was adjusted to 7.4 by adding NaOH, 5 mg of trypsin were added, and it was incubated for 6 h at 37 °C. The pepsin and trypsin digested gliadin (PT-gliadin) were boiled at 100 °C for 1 h and determined to be endotoxin-free by using the E-Toxate reagent (Sigma-Aldrich, 21020). The 33-mer peptide was supplied by Biomedal S.L. (Seville, Spain). The concentration of the antigens used for ELISPOT for both PT-gliadin and the 33-mer peptide was 100 mg/mL. Spot-forming Cells (SFCs) were counted using an automated ELISPOT reader (AID ELISPOT reader system; AID Autoimmun Diagnostika GmbH, Germany). The results were expressed as the Stimulation Index (SI), i.e., IFN-γ-SFCs counted in the presence of the PT-gliadin, or 33-mer/IFN-γ-SFCs detected in the presence of medium alone.

### 2.5. Quantification of the Gluten Immunogenic Peptides (GIP) in Stool Samples

All the participants were instructed to collect 2–4 g of stool sample in a sealed container, 6 days after the start of the challenge (day 6). Then, the stool samples were delivered within the next 2 h after deposition and stored at −80 °C until processing. All samples were identified and labeled using a random numeric code. Stool GIP concentration was determined by a sandwich ELISA iVYDAL In Vitro Diagnostics iVYLISA GIP Stool kit (Biomedal S.L., Seville, Spain) according to the manufacturer’s protocol [27]. The accuracy of this method in detecting gluten in stool has been reported previously, analytical sensitivity having limits of quantification of 0.08 μg GIP per g stool, and diagnostic sensitivity and specificity being 98.5% and 100%, respectively [28]. Each sample was analyzed in duplicate, and at least two different aliquots of each sample were tested on different days.

### 2.6. Statistical Analysis

To our knowledge, no studies of low gluten wheat in CD have previously been conducted; therefore, in preparation for larger observational studies and randomized controlled trials, we conducted a pilot study. Based on this, the calculation of statistical power was not considered necessary in this preliminary stage. Statistical software R version 3.6.1 was used to perform the data analysis and draw some of the graphs [29]. Figures were drawn using the software package Sigma Plot 11.0 (Systat Software, Inc., Point Richmond, CA, USA). The results of the quantitative variables were expressed using the median and the maxima. The Wilcoxon signed-rank test was used to analyze differences in the same subject among the different phases. A *p* < 0.05 was considered statistically significant.

## 3. Results

All recruited participants included 21 patients with CD on a GFD (13 female; median 38 years, range 19–64, SD of 11). Among them, one patient was excluded from the study because of consumption of gluten-containing foods, and the data were removed from the analysis. The study design was a blind randomized crossover gluten challenge (Figure 1) and was carried out at the Hospital of León, Spain. The randomization of the study was blinded to the patients and the clinicians involved in the trial; they did not know which bread they were consuming.

**Figure 2:**
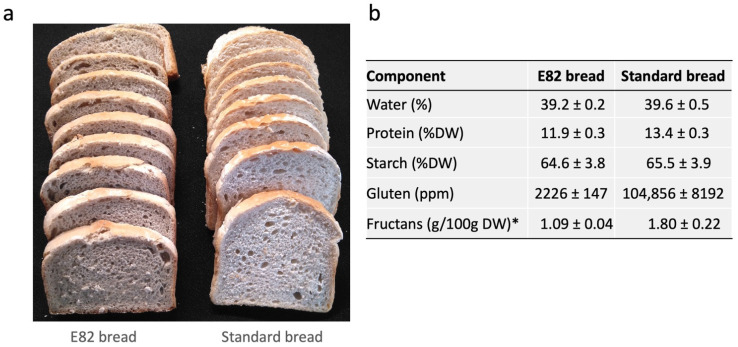
E82 and Standard bread types (**a**) used in this study and their main characteristics (**b**). Values in panel B represent the mean and standard deviation of four replicates. * Fructans were determined in white flour. DW, dry weight; ppm, parts per million.

Figure 2 shows the main characteristics of E82 bread and Standard bread used in this study. The volume of E82 bread was slightly lower than that of Standard bread. However, the texture and structure of the crumbs were similar between the two bread types. Both showed similar water content, and the starch content was also comparable between them. However, the protein content was significantly lower in E82 bread than in the Standard bread: 11.9 and 13.4% of dry weight, respectively. There were highly remarkable differences in gluten content between Standard bread (104,856 ppm) and E82 bread (2226 ppm). Fructan content, particularly related to symptoms in NCWS, was also determined, but in white flour, showing differences between Standard and E82 bread types.

### 3.1. IFN-γ Producing T Cells (ELISPOT)

**Figure 3:**
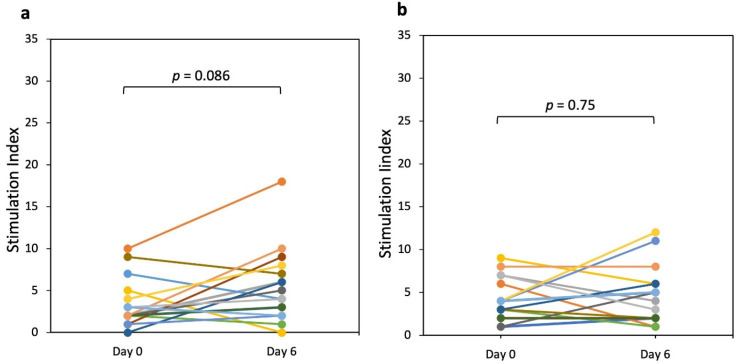
IFN-γ production elicited in peripheral blood after a 3-day challenge with E82 bread. Peripheral blood mononuclear cells (PBMCs) from patients with celiac disease (CD) were analyzed by an IFN-γ enzyme-linked immunospot (ELISPOT) assay before and after 3 days of E82 bread consumption (day 0 and day 6). The IFN-γ Spot-Forming Cells (SFCs) were measured and the Stimulation Index (SI) was calculated in response to (**a**) PT-gliadin (100 mg/mL) and (**b**) 33-mer peptide (100 mg/mL). The different coloured dots and lines represent the response of each patient. A non-parametric Wilcoxon signed-rank test was performed to compare the increment of responses between day 0 and day 6. SI: IFN-γ-SFCs counted in presence of the PT-gliadin or 33-mer peptide/IFN-γ-SFCs detected in the medium without antigens.

Figure 3 shows the gliadin-specific IFN-γ production elicited in PBMCs collected after a short (3 days) oral challenge with E82 bread. On day 6 the IFN-γ production elicited by PBMCs did not significantly increase (*p* = 0.086) in response to PT-gliadin (Figure 3a). On day 0 of the challenge, the median Stimulation Index (SI) value for PT-gliadin was 2.0 with a maximum value of 10. On day 6, the median SI value was 4.5 with a maximum value of 18 in one patient. Except for this patient, all patients showed comparable SI values between day 0 and day 6. We also determined the response to the 33-mer peptide with E82 bread between day 0 and day 6. No significant increase in IFN-γ production in response to the 33-mer peptide (*p* = 0.75) was found. The median SI response was 4.0 on day 0 (maximum value, 9) and 4.5 on day 6 (maximum value, 12) (Figure 3b).

**Figure 4:**
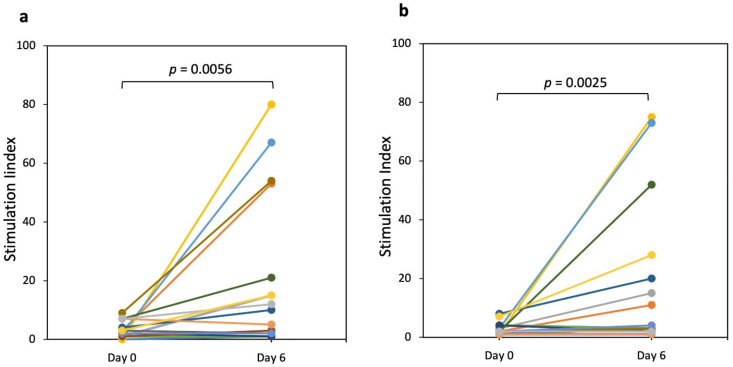
IFN-γ production elicited in peripheral blood after a 3-day challenge with Standard bread. Peripheral blood mononuclear cells (PBMCs) from patients with celiac disease (CD) were analyzed by an IFN-γ enzyme-linked immunospot (ELISPOT) before and after 3 days of Standard bread consumption (day 0 and day 6). The IFN-γ Spot-Forming Cells (SFCs) were measured and the Stimulation Index (SI) was calculated in response to (**a**) PT-gliadin (100 mg/mL) and (**b**) 33-mer peptide (100 mg/mL). The different coloured dots and lines represent the response of each patient. A non-parametric Wilcoxon signed-rank test was performed to compare the increment of responses between day 0 and day 6. SI: IFN-γ-SFCs counted in presence of the PT-gliadin or 33-mer peptide/IFN-γ-SFCs detected in the medium without antigens.

We also investigated the IFN-γ production after a short (3 days) oral challenge with Standard bread (Figure 4). The median SI values significantly increased (*p* = 0.0056) in response to PT-gliadin stimulation from 2.5 (maximum value, 9) to 8.0 (maximum value, 80) for day 0 and day 6, respectively. In addition, a significant increment (*p* = 0.0025) was observed between day 0 and day 6 after stimulation with the 33-mer peptide; the SI median value was 2.0 (maximum value, 8) on day 0 and 4.5 (maximum value, 75) on day 6 (Figure 4b).

### 3.2. Gluten Immunogenic Peptides (GIP) in Human Stool

**Figure 5:**
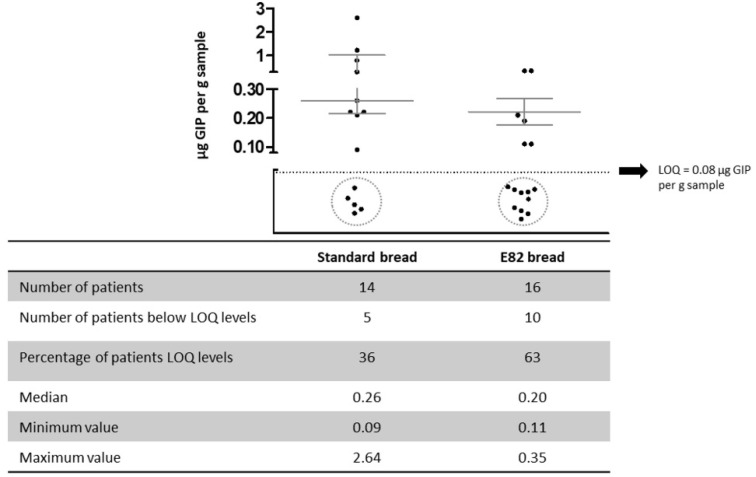
Concentration of gluten immunogenic peptides (GIP) in stools in patients after consumption of Standard and E82 bread types. The black dots represent the GIP values for each patient. LOQ, limit of quantification.

Figure 5 shows that after consumption of Standard bread, 9 out of 14 patients (64%) who provided the stool sample had detectable concentrations of GIP, with a median value of 0.26 μg GIP per g stool (range: 0.09–2.64). In contrast, after consumption of E82 bread, 10 out of 16 patients (63%) who provided the stool samples exhibited GIP content below the limit of quantification (LOQ) of the method. In patients in whom GIP was detected after consumption of E82 bread, they were at very low levels, with median values of 0.20 μg GIP per g stool (range: 0.11–0.35).

### 3.3. Gastrointestinal Symptoms

**Figure 6:**
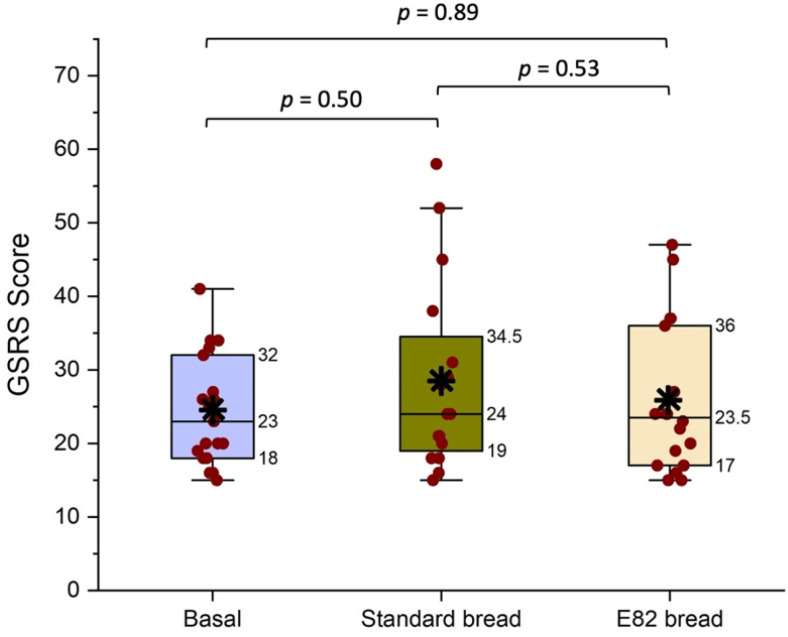
Gastrointestinal symptoms evaluated after short-term oral challenge with Standard and E82 bread types and compared to that of the gluten-free diet (GFD) by the Gastrointestinal Symptom Rating Scale (GSRS) questionnaire. The red dots represent the GSRS values for each patient. The black asterisk represents the mean. Values for Q1, median, and Q3 are indicated. The statistically significant differences were tested using the non-parametric Kruskal–Wallis test.

Gastrointestinal symptoms were evaluated at the end of the challenge with Standard and E82 bread types by completion of a GSRS questionnaire evaluating five parameters: indigestion, diarrhea, constipation, abdominal pain, and reflux. Results are shown in Figure 6, with higher values indicating a lower GI well-being. Results with Standard and E82 bread types were compared to that of the basal GFD (before starting the challenge). The median value (data for all subjects) during the basal GFD was 23 (range: 15–41) versus 23.5 (range: 15–47) after the consumption of E82 bread. The median value of GSRS during the challenge with Standard bread was 24 (range: 15–58). No significant differences were found between the GFD and E82 or Standard bread, nor between E82 and Standard bread (Figure 6).

## 4. Discussion

A GFD is the only treatment available for patients with CD because the symptoms usually disappear after the removal of gluten from the diet. Alternative cereals can improve the nutritional properties of the GFD but lack the baking properties that are characteristic of gluten-containing cereals [30]. Line E82, obtained by silencing RNAi, could be an outstanding alternative because its consumption by patients with non-celiac wheat sensitivity (NCWS) did not increase adverse clinical symptoms while providing a better gut microbiota profile [31]. Moreover, it has acceptable sensory parameters, an improved nutritional profile [32], and adequate bread-making properties [33].

Both E82 and Standard bread types contain comparable starch contents while the protein content (% dry weight) is 1.5% lower in E82 bread, despite the gluten content being reduced by 98%. As previously reported [34], the decrease in gliadins is compensated in this line by non-gluten proteins, thus preserving the total protein content in the flour. Therefore, considering the water and gluten content determined by the R5 antibody (mg/kg), it was estimated that patients consumed 12.67 and 0.27 g of gluten daily, while consuming Standard bread or E82 bread, respectively. However, these quantities may be different since the quantification of gluten by ELISA R5 has some drawbacks [35].

The effect of E82 bread was evaluated in vivo by monitoring the IFN-γ production by PBMCs and compared to that elicited by the consumption of Standard bread. We observed that the response of PBMCs was not significant after the consumption of E82 bread, even when the PBMCs were stimulated with PT-gliadin or with the 33-mer peptide, which has been included among the most GIP in patients with CD [5,36]. In contrast, Standard bread induced a significant increase in the PBMCs that reacted to both PT-gliadin and the 33-mer peptide. E82 bread diminished gluten-specific T cell responses compared to Standard bread that contained gluten. Although in this work PT-gliadin and the 33-mer peptide were not treated with tTG, significant differences could be seen between the two bread types. In this regard, differences for the IFN-γ production between digested gliadin with or without deamidation by tissue transglutaminase were reported [7,37]. However, Elli et al. [38] reported comparable IFN-γ levels in cultured duodenal biopsies from patients with CD exposed to PT-gluten and transglutaminase-treated gluten. Treatment with tTG could provide different responses in the IFN-γ production after stimulation with the two bread types, and therefore it needs to be considered in further clinical trials.

Analysis of stool samples confirmed that patients excreted much less GIP with E82 bread than they did with Standard bread. Moreover, the number in patients who showed GIP levels below the LOQ was twice as high after consumption of E82 bread compared to that of Standard bread. GIP content in patients with CD after E82 bread consumption was similar to that seen in patients with NCWS who had consumed bread also made with E82 flour [31]. In that study, patients with NCWS were eating up to 150 g per day of E82 bread for seven days. Previous studies found that GIP remained detectable in stools for up to 4 days, and the degree of gluten hydrolysis is strongly affected by the diet, and/or individual characteristics such as the gut microflora, among others [39]. In this study, stool samples were taken 3 days after the end of the challenge (day 6), while in the NCWS study, stool samples were taken the day after the end of the study.

The symptoms induced by the consumption of E82 bread were comparable to those of the GFD and not significantly different from those induced by Standard bread consumption either. These results are also comparable to those already reported for E82 bread consumption by patients with NCWS, where no differences in symptoms were observed between the GFD and E82 bread consumption [31]. However, this should be considered with caution in NCWS, as gluten does not appear to be the only culprit; other components such as FODMAPS, particularly fructans, may be responsible for gastrointestinal symptoms, and most patients with NCWS do not suffer deterioration when fed small amounts of gluten [40]. In the present study, fructan content was determined in white flour but not in the final bread types. Although there are differences between both types of flour, these amounts will be lower in bread as degradation of fructans has been studied in sourdough fermentation, showing reductions of up to 75% in the fructan content of bread in comparison to that of flour [41]. Despite significant differences between immunological activation induced by E82 and Standard bread types, symptoms were neither statistically significant between them or with those of the GFD. One possible explanation for this is that the threshold for inducing symptoms in patients with CD may be substantially different from that required to induce immunological activation, as suggested by [42], who reported that no differences in symptoms were found when comparing gluten treated with a mixture of two glutenases and placebo, although they found strong differences in immunological responses. Mandile et al. [43] also reported no symptoms in patients with CD during a 3-day challenge with consumption of 200 g of wheat baked goods per day. Anderson et al. [6] reported that few patients showed clinical symptoms after a short gluten challenge. In our study, patients with CD also consumed 200 g of bread daily. In the case of E82 bread, the gluten quantified by the R5 monoclonal antibody allowed us to estimate that the patients consumed about 257 mg of gluten daily. This amount is above 50 mg/day, which has been shown to induce an immune response in patients with CD [44]. However, previous proteomic studies on this E82 wheat line showed that it lacks the main epitopes that compose the 33-mer peptide [34], which could explain the lack of immune response observed in patients with CD.

This study provides preliminary evidence that E82 bread reduces disease-specific immunological activation following a short-term challenge in CD patients. The results re-ported in this study are close to real-life situations, with a consumption of 200 g of bread daily for three days, and a lack of an immune response in patients with CD. One of the limitations of the study is the sample size, which makes the power insufficient to draw strong conclusions. In addition, to obtain fully reliable results, stools should have been collected each day from day 3 to day 6. These finding encourage us to address future studies with E82 flour in longer tolerance multicenter trials, appropriate sample size calculation, and serological and histological analyses to confirm the absence of intestinal mucosal damage. In addition, strict monitoring of the diet of the participants is needed, as transgressions (voluntary or otherwise) could seriously mask the results. These findings also corroborate that the down-regulation of gliadins by the RNAi technology approach can provide low-immunogenic flour with the potential to be used as a raw material for baked goods.

Recently, in a relatively brief observation period of just 10 days, it was reported that two-thirds of participants had ingested and/or excreted gluten, and that the excretion kinetics are highly variable among individuals [11,45]. The authors demonstrated that most patients with CD can only accomplish a gluten-reduced diet, and the recommended strict GFD might not be maintained completely. These findings suggest that a strict GFD is difficult to achieve, and specific exposures are difficult to detect due to the variable time course of excretion. Thus, additional treatments are needed for this disease. If the results reported in this study are confirmed in large multicenter clinical trials, E82 flour could be suggested as an alternative to consumption by patients with CD. In particular, foods made using E82 flour may play a relevant role in the management of CD by improving the efficacy of the GFD. However, additional steps are necessary for such a product to reach the market, apart from the aforementioned clinical trials. The most important one is adequate regulation considering that it is not a drug but a food (Food and Drug Administration in the USA; European Food Safety Authority in the EU). This is not a trivial issue, since with the current regulation, foods containing less than 20 ppm of gluten, determined by ELISA techniques, are considered gluten-free in the USA and the EU, and E82 flour does not fit this. Another important aspect is that it is a GMO product, both in the EU and in the US, and therefore its regulation must follow the rules established by both agencies, both for cultivation and consumption. This aspect could be facilitated if the product is developed by CRISPR technology, which is currently viewed differently in the US and the EU.

## 5. Conclusions

This pilot study provides evidence that low-immunogenic flour can be obtained by the down-regulation of gliadins through RNAi technology in wheat. Our initial hypothesis was confirmed, as bread made from the RNAi E82 flour did not elicit an immune response after a short-term oral challenge. These results are promising for CD patients and, if these findings are validated in larger clinical trials, the E82 flour can be suggested as an alternative for celiac disease patients.

## Data Availability

Data described in the article will be made available upon request.
